# Methods for stability assessment of electrically conductive membranes

**DOI:** 10.1016/j.mex.2022.101627

**Published:** 2022-01-29

**Authors:** Mohamad Amin Halali, Charles-Franҫois de Lannoy

**Affiliations:** Department of Chemical Engineering, McMaster University, Hamilton, Ontario, Canada

**Keywords:** Electrically conductive membranes, Scratch resistance, Mechanical stability, Electrochemical oxidation, Polyvinyl alcohol, Carbon nanotubes, Glutaraldehyde, Succinic acid, Anodic potential, Electrified membranes

## Abstract

The surface properties of electrically conductive membranes (ECMs) govern their advanced abilities. During operation, these properties may differ considerably from their initially measured properties. Depending on their operating conditions, ECMs may undergo various degrees of passivation. ECM passivation can detrimentally impact their real time performance, causing large deviations from expected behaviour based on their initially measured properties. Quantifying these changes will enable consistent performance comparisons across the active and electrically conductive membrane research field. As such, consistent methods must be established to quantify ECM membrane properties. In this work, we proposed three standardized methods to assess the electrochemical, chemical, and physical stability of such membrane coatings: 1) electrochemical oxidation, 2) surface scratch testing, and 3) pressurized leaching. ECMs were synthesized by the most common approach – coating support ultrafiltration (UF) and/or microfiltration (MF) polyethersulfone (PES) membranes with carbon nanotubes (CNT) cross-linked with polyvinyl alcohol (PVA) and two types of cross-linkers (either succinic acid (SA) or glutaraldehyde (GA)). We then evaluated these ECMs based on the three standardized methods: 1) We evaluated electrochemical stability as a function of electro-oxidation induced by applying anodic potentials. 2) We measured the scratch resistance to quantify the surface mechanical stability. 3) We measured physical stability by quantifying the leaching of PVA during separation of a model foulant (polyethylene oxide (PEO)). Our methods can be extended to all types of electrically conductive membranes including MF, UF, nanofiltration (NF), and reverse osmosis (RO) ECMs. We propose that these fundamental measurements are critical to assessing the viability of ECMs for industrial MF, UF, NF, and RO applications.

• Anodic-oxidation was used to check the electrochemical stability of ECMs
• Depth of penetration resulted from scratch test is an indicator of the electrically conductive membrane coating's mechanical stability
• The leaching of the main components forming the nanolayer was quantified to assess the membranes’ physical stability

## Graphical abstract

**Figure:**
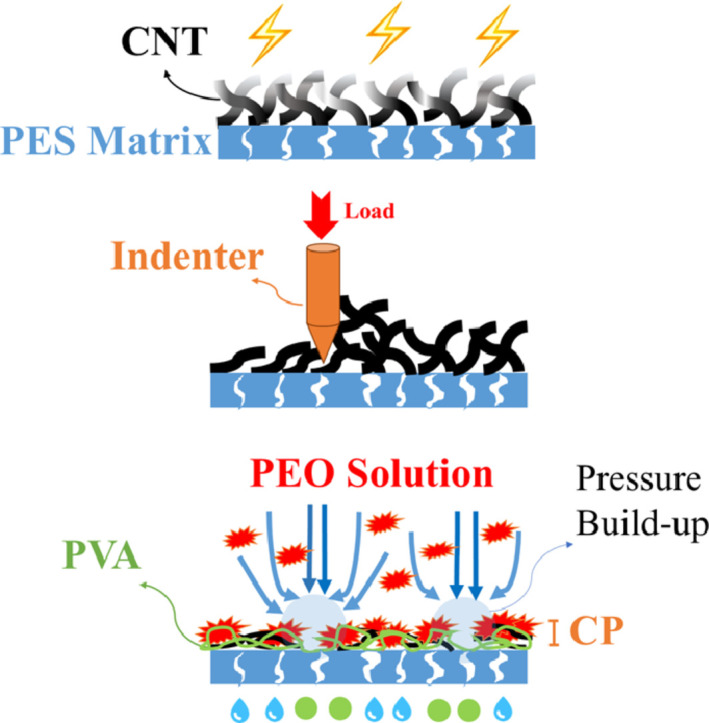

Specifications table

**Table:** 

Subject area:	Separation and Purification
More specific subject area:	Water and Wastewater treatment by conductive membranes
Method name:	Electro-oxidation, scratch test, and foulant separation to assess electrochemical, mechanical, and physical stability of ECMs
Name and reference of original method:	M. A. Halali, C. F. de Lannoy, Investigating the Stability of Electrically Conductive Membranes
Recourse availability:	The description of the details are provided in the manuscript [1]

## Method details

Membranes are the technology of choice for water and wastewater treatment due to their selectivity, smaller footprint, and low cost. Recently, electrically conductive membranes (ECMs) have gained great attention for their potential to improve the antifouling performance of membranes [2], [3], [4], [5], [6]. The first generation of ECMs are commonly made of CNT/PVA networks cross-linked with a cross-linking agent such as dicarboxylic acids or dialdehydes. These membranes can carry an externally applied electrical charge across their surface. The application of an electric potential to an ECM promotes various antifouling mechanisms at the membrane/water interface [6], [7], [8], [9], [10]. These antifouling mechanisms include electrostatic repulsion of like-charged foulants [11], electrochemical [12] and electrocatalytic [13] reactions, and gas generation [14]. ECMs have presented several advantages as compared to conventional membranes such as: (a) controllably target foulants at the membrane/water interface which makes them more effective than traditional bulk solution cleaning (biocide dosing, pH adjustment) [15], [16], [17], (b) use electrons, “clean reagents”, as antifouling mediators, making the process less chemical intense and easy to operate, which reduces the handling and storage costs of chemicals [18], [19], [20], and (c) can tailor their antifouling mechanisms by tuning the applied electrical properties (polarity, magnitude, and frequency). Therefore, the antifouling mechanisms can be tailored to exclusively match the application [7,17,21].

ECMs are synthesized with specific surface properties such as pore size, surface hydrophilicity, and surface conductivity to target different antifouling applications. The in-situ properties of membranes during the operation, however, may differ significantly with their initially measured properties. Membranes may be subjected to passivation during operation, leading to changes in their surface properties [22]. Membrane passivation would hinder their antifouling performance, impact their integrity, and increase the system energy consumption. For instance, the surface conductivity of ECMs allows for charge mobility across a surface, which in turn promotes antifouling mechanisms at the surface. Hence, the loss in conductivity would directly compromise the antifouling performance of such membranes. It is difficult to monitor the real time passivation of surfaces, and the in-situ membrane properties are often assumed to be unchanged from their initially measured properties. To date, the common characterization tests carried out after fabrication of membranes have failed to consider the operational impacts given that significant changes occur at the surface. The challenges of real time measurements (either direct or indirect) necessitate better assessment of surface properties.

Three types of ECM stability are the focus of the methods in this work. Electrochemical stability, mechanical stability, and physical stability. We propose specific methods to study these forms of ECM stability that should form the basis for all ECM assessments. Their importance and method of testing are described here:

*Electrochemical Stability*: We propose a method to assess the anodic stability of CNT-based membranes using a three-electrode electrochemical cell. ECMs are exposed to anodic, cathodic, alternating, or block currents (distinguished from alternating currents as anodic and cathodic currents applied sequentially at irregular intervals) depending on the foulant type and their application. Anodic current can permanently oxidize CNT-membranes [23], [24], [25]. CNT-corrosion decreases the surface conductivity critical for their antifouling performance. Many studies have reported the optimum potential required to promote interfacial mechanisms such as electrostatic repulsion, biofoulant inactivation, organic oxidation, or hydrogen peroxide generation [26], [27], [28], [29], [30], [31], [32]. However, the current-density correlated to the reported optimized potentials is significantly different from the real current density applied to the surface over the membrane's operational window. The real current density is often ignored due to lack of real time measurements and is therefore often overestimated.

*Mechanical Stability*: We propose scratch testing ECMs using a diamond tip to quantify the surface mechanical stability of membranes where the impact of surface chemistries is distinguishable. Cross-flow is the most common configuration in membrane-based systems regardless of the membrane type (microfiltration (MF), ultrafiltration (UF), nanofiltration (NF), or reverse osmosis (RO)) or membranes shape (flat sheet, hollow fiber, or spiral-wound). In such systems, membranes are constantly exposed to shear forces induced by tangential flow containing solid particles. In addition, cleaning procedures such as mechanical cleaning can damage the membrane surface. It is expected that surface damage induced by fouling, aging, scaling, or mechanical cleaning alter the surface properties of electrically conductive membranes [33], [34], [35], [36], [37]. Therefore, it is important to assess the mechanical stability of membranes used in separation-based systems. Mechanical stability of membranes is less explored as compared to other surface properties such as hydrophilicity, surface charge, and surface roughness.

*Physical Stability*: We propose a model foulant separation test to quantify the impact of concentration polarization on ECM polymer network instabilities. CNT-based membranes are most often composed of CNTs, organic polymers, and binders. The polymers and cross-linkers are added to take advantage of their hydrophilicity, stability, and film-forming ability. Polymers (PVA) and cross-linkers are attached via covalent bonds, electrostatic interactions, and H-bonding [38], [39], [40], however, a portion of PVA is loosely bound to the network and leaches under certain operating conditions (high transmembrane pressure, high foulant concentration) which results in changes in surface properties.

It should be noted that while the interactions between these various types of stability are not fully understood, the stability assessment methods discussed in this paper will allow researchers to compare membranes with different chemistries from mechanical, electrochemical, and physical perspectives. The described methods are focused on MF and UF membranes, however, they can easily be extended to NF and RO membranes as well.

## Materials and procedure

ECMs used in this study are synthesized following the same method published in our co-sbumitted work [41]. In short, pristine UF PES membranes were coated with CNT/PVA network cross-linked with either succinic acid (SA) or glutaraldehyde (GA). Unless otherwise mentioned, all ECMs contain 1 mg CNT and the CNT: PVA ratio was kept at 1:10 in all the membranes.

### Electrochemical Stability

A three-electrode electrochemical cell (Fig. S1) coupled to a potentiostat (Multi Autolab M204, Metrohm) was used to quantify the anodic oxidation state of the membranes through fixed potential chronoamperometry as well as cyclic voltammetry methods. In order to be able to record currents higher than 10 mA in response to high applied potentials, a booster (1A Metrohm) was coupled to the potentiostat. The CNT-based membrane, graphite sheet, and an Ag/AgCl electrode (Metrohm, operating range: 0-80°C) were used as the working electrode (anode), counter electrode (cathode), and reference electrode, respectively. All electrodes were placed in an electrochemical cell containing phosphate buffered saline (PBS, purchased from VWR) solution as the electrolyte. PBS solution contains 137 mM sodium chloride, 2.7 mM potassium chloride, and 10 mM phosphate buffer. The electrochemical cell was operated under continuously stirred conditions at 200 rpm. Supplementary tests were carried out to gain a better understating of the stability and CNT degradation mechanisms involved. The tests are described as follows:

### Fixed potential chronoamperometry

Anodic potential can oxidize CNT surfaces leading to a decrease in the current at the membrane surface. Fixed potential chronoamperometry can provide useful insights on the oxidation state of membranes over time. Fixed potential chronoamperometry reveals the magnitude and duration of the current that can be applied to the surface before the electrically conductive surface undergoes severe oxidation. In addition, the real current densities reported from this test can be used for closer predictions of antifouling mechanisms promoted by current density such as electrostatic repulsion force, gas generation, and radical generation. The efficient potential range for antifouling performance of ECMs has been reported to be in the range of 0 – 2 V. Under applied anodic potentials CNTs can corrode, as a result of various oxidizing mechanisms such as mechanical breakdown, oxidation of CNTs, and radical generation. A number of oxidation mechanisms are activated at potentials above 2 V, and have been explored for anti-fouling applications [42], [43], [44]. For these reasons, Fixed potential chronoamperometry tests were carried out by applying either 2 V or 4 V (with respect to the Ag/AgCl reference electrode) to the membrane for 240 min and 20 min, respectively, to explore the oxidation process. The time periods were chosen because the currents were shown to reach steady state over the test window. Hence, surfaces are expected to have minor oxidation changes beyond these time periods. Further, anodic current is applied often in different current types (direct or alternating current) or with different current duty ratios (ratio of pulsing time over one cycle). Therefore, the duration of the test represents a longer current window than would occur in normal operating conditions.

### Cyclic voltammetry

Cyclic voltammetry (CV) is a complementary test to fixed potential chronoamperometry. In this test, anodic potentials are applied to ECMs at a certain scan rate and for a number of cycles. It is expected that the magnitude of the current will decrease over each cycle until the membrane achieves a final oxidation steady-state. The rate of current drop over each cycle is a measure of the anodic stability over time. CV is an informative test as it exposes the membrane to a wide range of potentials that can induce different oxidizing mechanism. For the CV test, 15 cycles at a scan rate of 0.1 mVs^−1^ were applied to each membrane, with a scan range of 0 – 3 V relative to the Ag/AgCl electrode.

### Four-point conductivity meter

The conductive nanolayer in ECMs consists of a connected porous CNT network. Corrosion of CNTs leads to lower total conductivity of the network. As a result, it is expected that the decrease in ECM surface conductivity will lead to a decrease in their electrochemical conductivity. Therefore, the surface conductivity of membranes was measured before and after anodic oxidation at 4 V (with respect to the Ag/AgCl reference electrode) for 20 min. The surface conductivity was measured using a four-point probe conductivity meter. Before the test, membranes were fully dried at 60°C using an oven and cooled at ambient temperature. This instrument is equipped with two pairs of sharp needles arranged in a square configuration that are attached to adjustable arms. The needles are lowered onto the surface in such a way that they only contact the conductive surface and not the insulating polymeric support underneath. The probe applies a potential to the surface and calculates the sheet resistance by considering the resistance between different pairs of the needles. Before reporting the sheet resistance, the correct position of the needles should be confirmed by monitoring the resistance values between different pairs. If the CNTs are uniformly distributed across the surface, the resistance measured between all possible pairs of needles is expected to be within the same order magnitude. Any significant difference in the resistance between pairs of needles indicates that the needle probes have either not made complete contact with the conductive layer because they have not been lowered deep enough into the conductive coating, or that the coating is discontinuous.

### Total organic carbon analyzer

The breakdown of CNT structures because of passivation is expected to increase the organic carbon content in the electrochemical batch reactor. CNT destruction could occur from direct chemical oxidation of the CNT structure or from mechanical forces that are induced during the application of potentials. For example, the generation of hydrogen/oxygen gas during application of cathodic/anodic currents leads to formation of micro-bubbles. Micro-bubbles may get trapped inside the CNT network, and the subsequent increase in the size of these bubbles may cause mechanical breakdown of the CNT structures. The destruction of CNTs therefore increases the organic carbon content of the batch, which was measured before and after electro-oxidation at 4 V (with respect to the Ag/AgCl reference electrode) for 20 min using a total organic carbon analyzer (TOC, SHIMADZU). The data obtained from this method can monitor the extent to which electrical currents applied to the CNTs contribute to the mechanical breakdown of CNTs, which in turn leads to electrochemical instability.

### Fourier-transform infrared spectroscopy (FTIR)

Fourier-transform infrared spectroscopy (FTIR) is used to monitor the oxidation state of chemical structures. As mentioned, functionalized CNT, PVA, and cross-linkers are bound in an electrically conductive membrane (ECM) network through covalent bonds, electrostatic interactions, and H-bonding. The intensity of C-O, C=O, and O-H bonds associated with such components is expected to change over the oxidation period. As a result, FTIR allows us to identify the changes in their chemical bonds.

### Micro scratch tester

Scratch testing can be used to assess the mechanical stability of the electrically conductive coatings of ECMs. Scratch testing can be carried out on three different scales: macro, micro, and nano depending on the thickness of the electrically conductive coating. Thin metallized layers, for example, should be tested with a nano-scratch tester, while thicker CNT or rGO layers should ble evaluated with a micro-scratch tester. In this study, a micro scratch tester manufactured by Anton Paar (Revetest scratch tester) was used. The specifications of this device are shown in Table 1. Scratch testing consists of three steps: 1. Pre-scan – The physical state and topography of the surface is monitored before applying any force, 2. Scratching – The surface is scratched using predefined settings such as scratch path, speed, and type, and 3. Post-scan – The physical state and topography of the surface is monitored after scratching and damaging the surface. It should be noted that pre-scan and post-scan should be carried out under the lowest pressure possible to minimize any additional damage to the surface beyond that caused by the scratch-test. We defined the correct scratch settings based on instructions of the manufacturer for polymeric coatings as well as performing different scratch trials prior to the real scratch test. These settings include the scratch type (constant, progressive, or incremental), scratch length, scratch speed, applied normal load, indenter type, indenter shape (Spherical, Berkovich, and Vickers), indenter radius, and indenter angle. The manufacturer recommends a scratch testing with a Rockwell diamond indenter to evaluate the resistance of polymeric or ceramic materials. The critical load (loads at which material failure occurs), scratch length, and scratch speed must be defined by the user depending on the type of the material. The settings used in our study for each of the parameters in Table 1 are provided in Table 2. These settings have been identified as optimal for membrane surfaces coated with carbon nanotubes, as demonstrated in this study. We recommend all researchers to use these settings to standardize scratch-testing across graphitic nanomaterial ECMs. ECMs composed of metallized surfaces or conductive polymers will require different settings. The micro scratch tester reports parameters such as depth of penetration, normal force, tangential force, acoustic emission, and friction coefficient. Acoustic emission is used to monitor the material failures including cracks and voids across the network in non-destructive testing [45]. Friction coefficient, μ, is the ratio of tangential force over normal force and is mostly μ< 1. A rupture in surface would lead to friction coefficients μ > 1. Depth of penetration of the indenter over the scratch length was used as an indication of the mechanical stability. Three scratches should be performed on each surface to validate the reproducibility of the results.

**Table 1:** Specifications of the micro scratch tester (Anton Paar).

Parameter	Value
Maximum Normal load	100 N
Normal load resolution	0.1 mN
Maximum friction load	100 N
Friction force resolution	0.1 mN
Maximum scratch length	70 mm
Scratch speed	0.4-600 mm/min
Maximum depth	1000 µm
Depth resolution	0.5 nm

**Table 2:** Micro scratch settings used.

Settings	Value
Indenter characteristics	
Type	Rockwell
Shape	Spherical
Material	Diamond
Radius	200 µm
Tip angle	90 °
Serial number	AJ-259
Scratch characteristics	
Type	Constant
Normal load	0.5 N
Scratch length	1 mm
Scratch speed	5 mm/min

### Model foulant separation test

The coatings in electrically conductive membranes (ECMs) contain nanoparticles and/or polymers. This is true for ECMs composed of graphitic nanomaterials, conductive polymers, and metal thin films. Loosely bound molecules may leach from such coatings leading to surface instabilities. Surface passivation induced by leaching of molecules may cause alterations in surface properties, therefore, it is important to quantify the chemical stability of ECMs. To this end, we recommend challenging the surface with model foulants in a realistic separation process and evaluating the impact of concentration polarization on the stability of the conductive coating components. A filtration process allows for model foulants to accumulates at the surface leading to formation of a concentration polarization (CP) layer. Pressures build up locally at the CP layer which can dislodge chemically unstable molecules. Leached molecules should be measured and quantified in the permeate to evaluate the physical stability of the conductive nanolayer. Our suggestion is to use polyethylene oxide (PEO) as a model foulant. PEO is commonly used to identify the molecular weight cut off (MWCO) of membranes, it is inexpensive, and it is widely available in a range of molecular weights making it effective for inducing concentration polarization in MF, UF, NF, and RO membranes. While the MWCO of a membrane is defined by the molecular weight of PEO particles that are rejected at >90% by the membrane, in our pressurized stability test, PEO is used to induce concentration polarization. As such, researchers should choose a PEO particle size that is effectively rejected from the membrane > 99.5%. Using this size of PEO, it is expected that these polymer particles will accumulate at the surface, form a strong CP layer, and induce strong local pressures that may force any loosely bound molecules out of the conductive layer.

We conducted a polyethylene oxide (PEO) separation test to evaluate the physical stability of the nanolayer with the experimental conditions provided in Table 3. The carbon content in the permeate was quantified using a total organic carbon analyzer (TOC, SHIMADZU). For example, our pristine ultrafiltration (UF) polyether sulfone (PES) membranes, with nominal pore size of 0.03 µm efficiently reject 2 MDa polyethylene oxide (PEO) particles. Under the assumption of a robust stable nanolayer, ECMs are expected to have an equal or higher rejection performance as compared to pristine PES membranes due to the presence of the extra separation barrier coating the support membrane (in our case a porous CNT network). If the nanolayer is unstable, the carbon content in the permeate will be higher than that found in pristine PES membranes due to polymer (or nanomaterial) leaching. The degree of instability can then be correlated to the magnitude of leaching.

**Table 3:** Operating conditions of the separation test.

Parameter	Value
Membrane Nominal pore size	30 nm
Model foulant	Polyethylene oxide
Foulant size	2 MDa
Feed concentration	50-250 ppm
Transmembrane pressure	10-100 psi
CNT mass loading in nanolayer	1-3 mg

Leaching from the ECM can be quantified by measuring the change in the organic carbon content in the permeate. The organic carbon content in the permeate will be composed of the polymer that leached from the ECM, as well as the small fraction of PEO that may pass through the membrane. If leaching is measured from the membrane under PEO testing, then a series of control separation tests should be performed to identify the sources of carbon content in the permeate as follows:

1. *Separation test on support membranes*: This test provides an average value for permeated PEO particles through the support membranes (e.g. UF PES membranes).
2. *Separation test on the support membrane exposed to the same conditions used to fabricate the ECM*: ECMs are often fabricated using high temperature curing, exposure to acids or bases, or other harsh processing conditions that could impact the support membrane. These controls conditions identify if such parameters contribute to changes in the rejection performance of the support membrane.
3. *Pure water flux on PES membranes and ECMs*: This is a negative control test to ensure that applied pressure during pure water permeability does not cause physical instability.
4. *Separation test on individual components of the ECMs*: ECMs are often composed of nanomaterials, polymers, binders, cross-linkers, and other additives. To identify the source of the leachates may require making ECMs with each of these components separately. For example, in this study we evaluated the impact of concentration polarization on networks composed of CNTs alone, CNT with PVA, and CNT with PVA cross-linked with glutaraldehyde. Each of these control conditions help identify which component was contributing to the permeate leachate.

In our example, local pressure build-up induced by concentration polarization induced leaching of loosely bound polymeric particles. As a result of concentration polarization, PEO particles partially block the surface leaving fewer pathways for water permeation. Local pressure increases in the open pores led to leaching of unreacted and unstable polymers (e.g. PVA). In order to quantify the degree of leaching, the physical instability should be tested at different operating pressures, different feed concertation, and at thicknesses of the nanolayer as provided in Table 3.

## Method validation

### Anodic oxidation

**Fig. 1:**
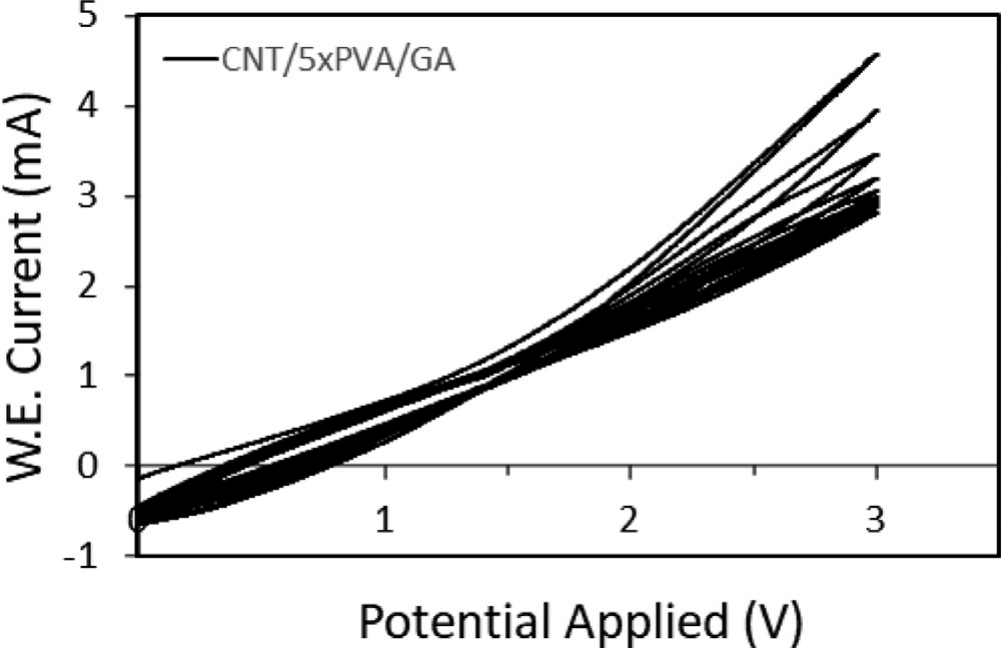
Cyclic voltammetry. ECM contains 1 mg CNT mass loading. The conductive ECM, graphite sheet, and an Ag/AgCl electrode were used as the working electrode (anode), counter electrode, and reference electrode, respectively. The scan range was 0 – 3 V at a scan rate of 0.1 mVs^−1^.

**Fig. 2:**
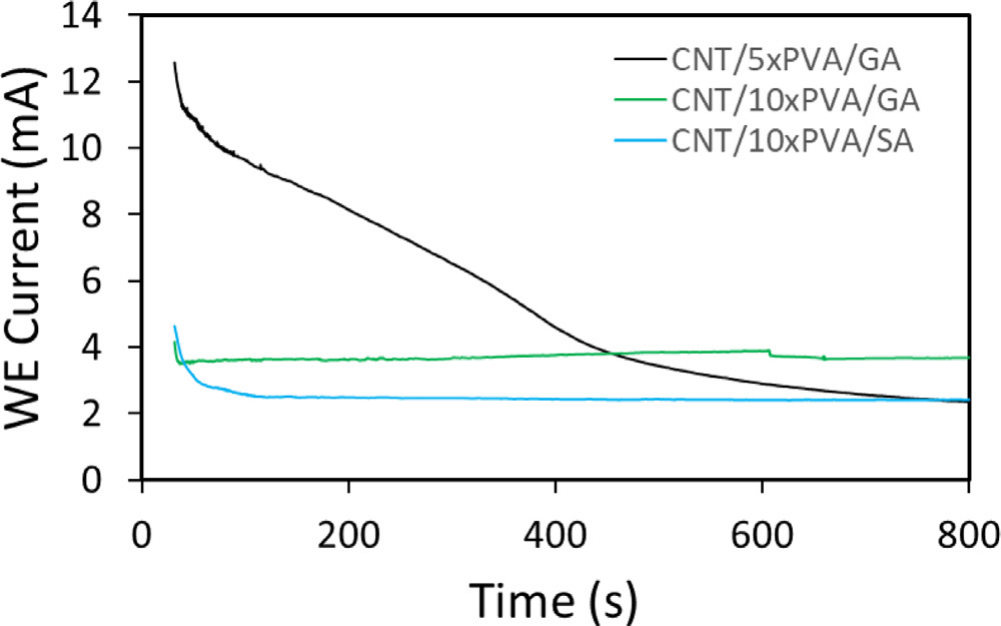
Fixed potential chronoamperometry for membranes with different chemistries containing CNT, PVA, and cross-linkers (glutaraldehyde, GA, and succinic acid, SA). Trials were conducted for each network as follows. ECM, graphite sheet, and an Ag/AgCl electrode were used as the working electrode (anode), counter electrode, and reference electrode, respectively. The total cell potential was 4 V. All ECMs contain 1 mg CNT.

Fig. 1 shows the results of cyclic voltammetry applied on an ECM containing 1 mg CNT, 5 mg PVA, and glutaraldehyde (GA) as the cross-linker (CNT/5xPVA/GA). The conductivity of the membrane decreases over 15 cycles, as shown in Fig. 1. The drop in conductivity is greater in the first 5 cycles indicating a fast oxidation rate while it is lesser in the next 10 cycles indicating steady state oxidation. These results can be correlated to findings in Fig. 2 where fixed potential chronoamperometry was applied. In Fig. 2, the conductivity of an ECM with the same chemistry as in Fig. 1, (CNT/5xPVA/GA), follows the same trend in oxidative change. Initial fast corrosion leads to a steady state plateau. The fixed potential chronoamperometry method is used to quantify the electrochemical stability of ECMs containing different types and amounts of cross-linkers. A comparison among different ECMs is provided in Fig. 2. Membranes containing 1 mg of CNTs, 10 mg PVA, and glutaraldehyde (CNT/10xPVA/GA) showed higher final conductivity even thought they had a lower initial conductivity. The lower initial conductivity is due to the higher amount of electrically insulating PVA (10 mg vs 5 mg) within the network. Interestingly, the greater stability of membranes with higher PVA may be attributed to oxidative protection of the CNTs by the polymer.

**Fig. 3:**
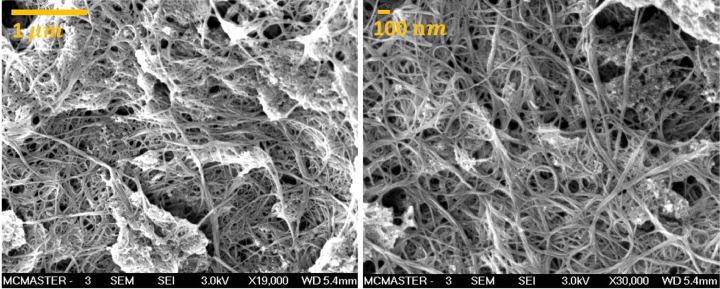
SEM images of CNT-ECM: (on the left) before, and (on the right) after electrolysis working as an anode under 2 V for 180 min. ECMs contain 1 mg of CNT.

Detailed experiments as well as the results of surface conductivity, mechanical conductivity, and chemical bonding state of membranes with different networks are provided in the original manuscript [1]. ECM conductivity measurements before and after surface oxidation (not shown here) revealed that the ECM surface conductivity was lower for all ECMs after oxidation, supporting the results from chronoamperometry. The mechanical breakdown of CNT structures was quantified using a total organic carbon analyzer and found to be insignificant. FTIR analysis, found in the supplementary information of the original manuscript [1], was conducted to monitor the presence of chemical bond formation in response to a current applied to the ECMs. The presence of C=O bonds increased during electrochemical oxidation. We hypothesize that this increase in C=O bond formation is a result of acetylation reactions between the cross-linker (GA) and PVA. It is worth noting that no noticeable damage was observed on CNT structure after exposure to 3 h anodic oxidation (2 V), demonstrated by scanning electron microscopy (SEM, Fig. 3).

### Scratch test

**Fig. 4:**
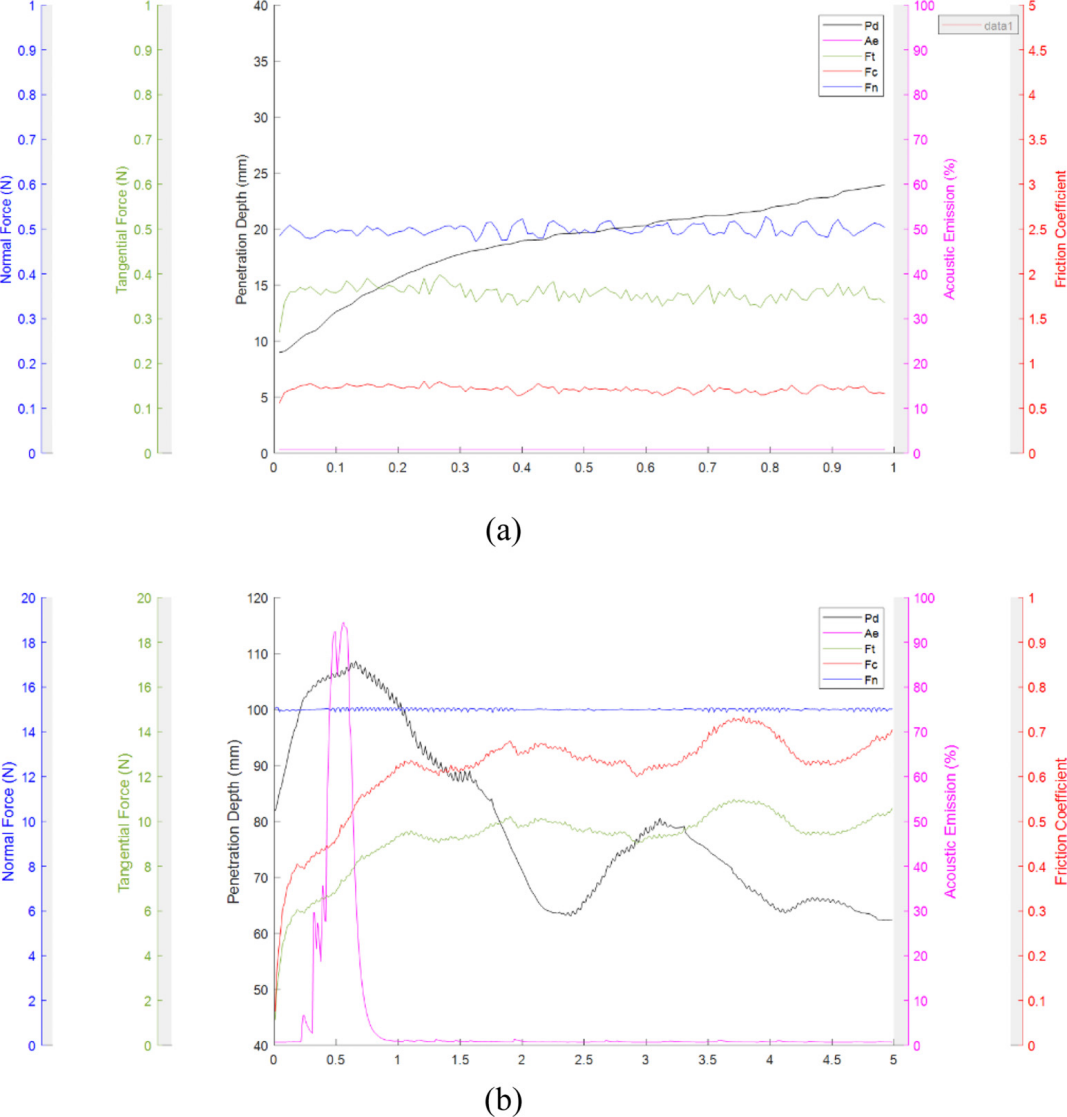
The mechanical stability of an ECM measured in the micro-scratch test with different input settings. The settings are as follows: (A) The tip of radius 200 µm applied a normal force of 0.5 N and the scratch length and scratch speed were 1 mm and 5 mm/min, and (B) The tip of radius 100 µm applied a normal force of 15 N and the scratch length and scratch speed were 5 mm and 5 mm/min.

The results of the micro scratch test on an electrically conductive membrane with the correct settings is presented in Fig. 4a. As indicated, the depth of penetration increased over the scratch length which shows the degree of instability. Membrane rupture leads to data that cannot be quantifiably analyzed; it is important to identify the critical operational settings to avoid this outcome by running control tests. The settings include indenter radius, applied normal force, scratch length, and scratch speed. Thus, membranes were purposefully ruptured to demonstrate the difference between and successful and failed scratch test. An example of a failed test is presented in Fig. 4b. More results of failed tests can be found in the supplementary information.

### Rejection test

The results of induced concentration polarization were discussed in detail in the original manuscript [1]. As explained in the Methods section, a series of control tests were conducted to assess the stability of the nanolayer followed by deconvolution of leaching sources as follows:

- 2 MDa PEO separation test on pristine PES membrane:
  ○ Negligible carbon content in the permeate side
- 2 MDa PEO separation test on PES membrane exposed to the processing conditions used in making the ECMs, including high temperature curing and acidic solutions:
  ○ Negligible carbon content in the permeate side
- Pure water flux on PES membranes and ECMs:
  ○ Negligible carbon content in the permeate side
- 2 MDa PEO separation test on ECMs containing PVA:
  ○ High carbon content observed in the permeate side

A summary of leachate values quantified from different rejection tests is provided in Table 4 [1,41]. In short, UF PES membranes fully rejected 2 MDa PEO particles (99.6%). However, the measured permeate carbon content was high in membranes composed of CNTs cross-linked with PVA formed on a support UF PES membrane. These findings led us to speculate that the nanolayer is physically unstable under concentration polarization conditions, or high pressure conditions, and that the PVA could leach through the membrane under certain conditions. It should be noted that pure water flux at the same pressures as applied during MWCO testing did not lead to an increase in the permeate carbon content. As a result, we hypothesize that local pressure build-up induced by concentration polarization was responsible for PVA leaching. PEO separation tests under either a higher PEO concentration (250 ppm compared to 50 ppm) or under a higher transmembrane pressure (TMP, 100 psi compared to 10 psi) were conducted to confirm our hypothesis. It was observed that either a higher feed concentration or a higher TMP led to higher leaching. To alleviate the local pressure build-up, thicker CNT layers (containing three times higher CNT mass loading) were synthesized which could extend the concentration polarization layer. The results revealed that membranes with thicker nanolayers resulted in less leaching (by up to 50% less leaching) due to an extended concertation polarization layer.

**Table 4:** Leachate values measured by different rejection tests.

Membrane	CNT mass loading (mg)	PVA mass loading (mg)	PEO Concentration (mg/L)	Pressure (psi)	Fabrication Temperature (°C)	Fabrication pH	Leachate (mg/L)
**PES**	-	-	250	10	25	7	5.87
**PES**	-	-	250	10	25	3	9.88
**PES**	-	-	250	10	100	7	3.24
**PES**	-	-	-	10	25	7	0.00
**ECM**	1	-	250	10	25	3	2.01
**ECM**	1	10	-	10	25	7	0.96
**ECM**	1	10	-	100	25	7	1.44
**ECM**	1	10	250	10	25	7	41.25
**ECM**	1	10	250	100	25	7	84.97
**ECM**	1	10	50	10	25	7	24.02
**ECM**	1	10	50	100	25	7	53.53
**ECM**	3	30	250	10	25	7	36.10

## Declaration of Competing Interest

The authors declare that they have no known competing financial interests or personal relationships that could have appeared to influence the work reported in this paper.

## Appendix Supplementary materials

Image, application 1
