# Supplementary material for: Methods for stability assessment of electrically conductive membranes
Source: MethodsX. 2022 Jan 29;9:101627. doi: 10.1016/j.mex.2022.101627 (PMC8844790; doi:10.1016/j.mex.2022.101627)
Supplement: Supplementary file 1 [file mmc1.docx]

Methods for stability assessment of electrically conductive membranes

**Mohamad Amin Halali, Charles-Franҫois de Lannoy**

**Department of Chemical Engineering, McMaster University, Hamilton, Ontario, Canada**


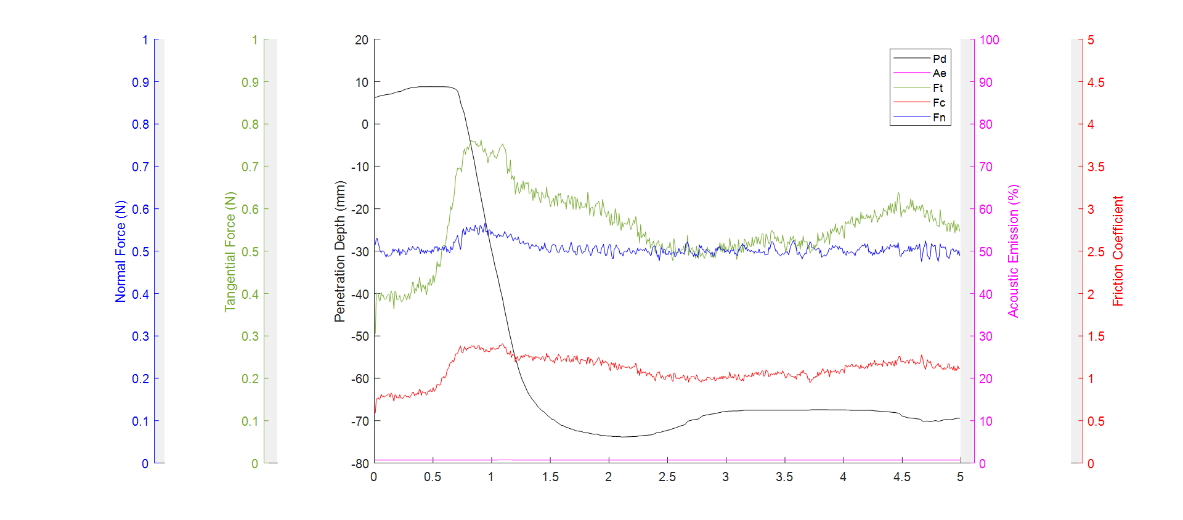


(a)


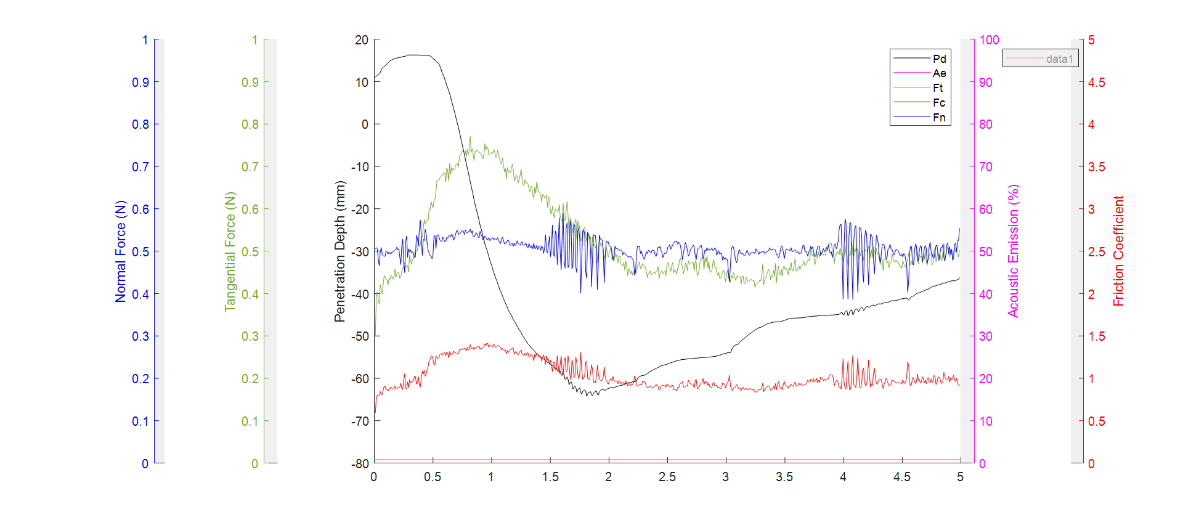


(b)

Fig. S1. The mechanical stability of an ECM measured in the micro-scratch test with different input settings. The settings are as follows: (A) The tip of radius 100 µm applied a normal force of 0.5 N and the scratch length and scratch speed were 5 mm and 5 mm/min, and (B) The tip of radius 200 µm applied a normal force of 0.5 N and the scratch length and scratch speed were 5 mm and 5 mm/min, respectively.
